# Wearing face masks when no longer mandatory: An exploratory study about attitudinal and psychological health factors in a large Italian sample

**DOI:** 10.1371/journal.pone.0314607

**Published:** 2025-03-04

**Authors:** Daniela Di Riso, Silvia Spaggiari, Giulia Calignano, Paola Rigo, Marina Miscioscia

**Affiliations:** Department of Developmental and Socialization Psychology (DPSS), University of Padua, Padova, Italy; University of Glasgow, UNITED KINGDOM OF GREAT BRITAIN AND NORTHERN IRELAND

## Abstract

Throughout the Covid-19 pandemic, face masks emerged as a critical tool to control the virus transmission. While previous studies have investigated the positive and negative attitudes towards the use of face masks when mandatory, our study looks at a different phase: the era of discretionary mask use. Our investigation reached Italian participants (1151), aged 18-64, who underwent an online survey. The study explored the associations between demographic, attitudinal, psychological, and emotional factors that may be involved in the behaviour of wearing a face mask. Then, by using generalized mixed effects models, we explored the predictive role of those factors selected via backward elimination starting from a full model and selecting the model with the best goodness of fit balanced with complexity, and higher explained variance. Specifically, our exploratory study expected that emotional experience aroused by wearing a face mask, negative affectivity, Covid-19-related fear, and anxiety might be significant predictors of face mask wearing. However, only the emotional experience of feeling a sense of care and protected while wearing a face mask, and Covid-19-related fear were significant predictors of face mask-use. We discussed the importance of considering attitudes involved in compliance with healthy behaviours to guide future health interventions.

## Introduction

Why do some people wear masks even though the Covid-19 emergency has passed and health protective equipment is no longer necessary? A deeper understanding of this phenomenon could be fundamental for new lines of research that explore the underlying mechanisms of adherence to healthy behaviors. Additionally, it could contribute to understanding the psychological processes involved in the development of hyper-inflexibility and adherence to protective health behaviors.

The world has been struggling against the Covid-19 pandemic in the last few years, with still evident impacts. The World Health Organization recommended using face masks to limit the spread of the virus in June 2020 [1]. Since then, many countries increasingly started to strictly require face mask-wearing as a way of protecting others from contracting the virus [2]. Indeed, literature widely studied positive and negative attitudes toward face mask-wearing in 2020, when it was mandatory in most countries (e.g., [3]). Nevertheless, there is still a paucity of knowledge about how these attitudes evolve when face masks are not formally required, despite the ongoing status of the pandemic.

In fact, the limited research evidence on face mask wearing when not mandatory suggests that individual and contextual factors, including low emotional well-being, high levels of Covid-19-related anxiety and psychoticism, and high levels of trust in healthcare professionals may play a role in the decision to wear a mask in closed environments [4]. Other evidence has highlighted that situational contexts, such as public transport vs supermarkets, may influence the use of face masks when no longer mandatory [5]. Given the current state of research on this topic, more research is needed to clarify the role of socio-psychological factors in the use of facemasks when non-mandatory.

Among the factors associated with the behaviour of wearing face masks when mandatory, the emotional experience seems to represent a key variable useful in predicting face mask wearing. It is worth mentioning that, based on terror management theory, feeling that one’s life is at risk, drives health-protective behaviours to reduce the threat and increase self-esteem. Moreover, research shows that experiencing both positive and negative emotions (e.g., feeling controlled, weak, scared, stupid, brave, caring, strong, and protected) may influence personal compliance with health protection measures during the pandemic [6,7,8,9]. A review of the literature on the aforementioned emotions of feeling controlled, weak, scared, stupid, brave, caring, strong, and protected reveals intriguing findings during the period of mandatory face mask wearing. For example, the social pressure not to show weakness (in men) [10, 11], high levels of feeling controlled and limited in personal freedom [11], and low levels of feeling brave and strong [12] are associated with refusal to use a facemask. In addition, the belief that wearing a facemask makes people look “silly” is associated with facemask refusal [13]. Conversely, feeling protected from contagion may increase facemask use [12,14], as well as social responsibility, empathy and prosociality for others [14, 15,16]. Findings on emotional experience, when integrated with findings on socio-psychological factors, may help to understand similar behaviours in the context of non-mandatory face mask use.

Indeed, associated with the mandatory use of face masks, research has identified a number of individual factors, including demographics, attitudes and psychological aspects, that influence the adoption of protective behaviours in different contexts. In terms of demographic factors, individuals belonging to the higher age groups and educational levels, were more likely to use face masks [7,17]. However, one study found demographic variables and face mask-wearing to be unrelated [13]. Among the most studied attitudinal factors, reactance seems to be associated with negative attitudes toward face mask-wearing, while fear of Covid-19 contagion, trust in healthcare professions and resilience may play key roles in following health-protecting behaviors [18,19,20,21,22,23]. Moreover, attitudes toward physical touch have been taken into account during the pandemic (e.g., [24]), but not yet in relation to people’s choice to wear or not a face mask. Individuals may have varying attitudes towards physical touch, which can influence their behavior in maintaining distance from others and thus contribute to self-isolation through mask-wearing. Both mask usage and social distancing became protective behaviors to safeguard personal health and that of loved ones [24]. Finally, regarding psychological factors, it has been reported that anxiety and depression levels, which significantly raised in adults during the early pandemic, could have had a negative impact on adherence to Covid-19 health behaviors [25]. Personality traits also seemed to be predictors of recommended health behaviors during the pandemic [26]. A recent article on Italian undergraduate students found that people with high internalizing personality traits such as detachment, negative affectivity, and psychoticism, were more likely to experience depression and stress symptoms, that in turn were linked to less compliance with health protective measures [27].

Given the lack of knowledge about the factors that may influence the use of face masks when they were no longer mandatory, this preliminary study aimed to explore the role of a wide range of variables in predicting that behavior in Italy, in a timeframe where the Government no longer required face masks but where the pandemic was not yet over. We focused on the mentioned demographic, attitudinal, psychological, and emotional factors, by using a Backward Elimination approach. In light of the literature in the field, we expected that emotional experience elicited by wearing a face mask, negative affectivity as a stable personality trait, situational factors such as fear of Covid-19, and anxiety might be significant predictors. Specifically, we expected that negative emotional experiences would be linked to less use of face masks, whereas positive emotional experiences would be linked to greater use. Furthermore, we expected negative affectivity to predict less adherence to face mask use [27]; coherently, high levels of anxiety may be detrimental to the adoption of healthy behaviors [25], whereas fear of Covid-19 may lead people to increase face mask use to protect themselves from the contagion [21].

## Materials and Methods

### Participants

We collected data from 1268 participants. Inclusion criteria were age 18-64 years and online survey full completion; after applying the inclusion criteria, 117 participants were excluded. The final sample included 1151 adult participants (age M =  32.4 years; SD =  12.1).

### Procedure

Data were collected from June 30, 2022, to September 30, 2022. In Italy, from 16 June to 30 September, face masks were only required on public transport and in healthcare services. Participants were recruited through social media, local flyers, and word of mouth. Participants were fully briefed and gave their informed consent to the procedure that was accepted by the Ethics Committee on Psychological Research Areas of the University of Padua (no. 4731/2022). This research was conducted in accordance with the Declaration of Helsinki. Finally, participants were informed that their data would be analyzed aggregated and anonymously. The protocol consisted of an online interview implemented through the Qualtrics online platform and accessible via a web link. The initial screen of the interview displayed the informed consent form, and the participant could proceed to subsequent pages only after providing written consent. The interview consisted of an ad hoc socio-demographic interview and a psychological assessment which included the following questionnaires: Multidimensional Assessment of Covid-19-related Fears (MAC-RF), Personality Inventory for DSM-5 Personality Disorders Short Module (PID-5-BF), Generalized Anxiety Disorder Scale-7 (GAD-7), and Patient Health Questionnaire-9 (PHQ-9). The current database is part of a broader research project aimed to investigate the impact of several psychological and social factors on the use or non-use of face masks during the Covid-19 pandemic [4].

### Sociodemographics and Self-report Questionnaires

The ad hoc Socio-Demographic Interview assesses demographic characteristics (e.g., age, sex assigned at birth, nationality, relationship status, education level), attitudes (e.g., attitudes toward physical touch, resilience, reactance, trust in healthcare professions), emotional experience associated with the thought of wearing a face mask (e.g., feeling controlled, weak, scared, silly, brave, caring, strong, protected), and the Covid-19 experience (e.g., Covid-19 vaccination, having had Covid-19). More specifically, the items used to assess resilience, reactance, and trust in healthcare professions were drawn from the Brief Resilient Coping Scale ([28]; “No matter what happens to me, I believe I can control my reactions”), a study by Dillard & Shen ([29]; “I get angry when my freedom of choice is restricted”), and a work by Mallinas et al. ([19]; “I trust the health professions and their indications ”). Moreover, the attitudes toward physical touch were assessed (e.g., I often find touching or being touched an intrusive gesture). The items were rated on a 5-point Likert scale from 1 (I strongly disagree) to 5 (I strongly agree). Moreover, participants were asked if they were still using a facemask in closed environments despite they were no longer mandatory. The answers were 1 for yes and 2 for no.

Multidimensional Assessment of Covid-19-Related Fears (MAC-RF; [30]) is an 8-item scale aimed to assess relevant Covid-19-related fears experienced during the past week, derived from an interplay of cognitive, bodily, behavioural, and interpersonal features. Each item is rated on a five-point Likert scale ranging from 1 (Very unlike me) to 5 (Very like me); the total score can be between 0 and 32, and a higher score indicates greater fears related to Covid-19. The scale has good internal consistency (Cronbach’s alpha = .84), satisfactory split-half reliability (Spearman-Brown r = .78), and in the present study, Cronbach’s alpha was α=.80.

Personality Inventory for DSM-5 Personality Disorders-Brief Form (PID-5-BF; [31]) is a 25-item scale to assess pathological personality traits, namely, negative affectivity, detachment, antagonism, disinhibition, and psychoticism (e.g., “People would describe me as reckless”). Each item is rated on a four-point Likert scale from 0 (never) to 3 (always); the total score can vary from 0 to 75, and a higher score indicates greater dysfunction in the specific personality trait domain. The scale has good internal consistency, and in the present study, Cronbach’s alphas for negative affectivity, detachment, antagonism, disinhibition, and psychoticism were respectively α=.59, α=.62, α=.62, α=.71, α=.69.

Generalized Anxiety Disorder Scale - 7 (GAD-7; [32]) is a 7-item scale designed to assess worry and anxiety symptoms during the last 2 weeks (e.g., “Worrying too much about different things”). Each item is rated through a four-point Likert scale from 0 (not at all) to 3 (nearly every day); the total score can vary from 0 to 21, and a higher score indicates greater severity of anxiety. The scale has excellent internal consistency (Cronbach’s alpha = .92), test-retest good reliability (intraclass r = .83), and in the present study, Cronbach’s alpha was α = .90.

Patient Health Questionnaire - 9 (PHQ-9; [33]) is a 9-item scale that assesses the severity of depressive symptoms during the last 2 weeks (e.g., “Little interest or pleasure in doing things”). Each item is rated on a four-point Likert scale from 0 (not at all) to 3 (nearly every day); the total score on the test can vary from 0 to 27, and a higher score indicates greater severity of depression. The scale has excellent internal consistency (Cronbach’s alpha = .89), and in the present study, Cronbach’s alpha was α=.87.

### Statistical Analysis

Data were analyzed using R software [34], specifically employing Generalized Linear Mixed-effects (GzLM) modeling to estimate relations among the variables. GzLM accounted for fixed effects (i.e., variables of interest) while specifying the residual distribution family, i.e., binomial, to address assumptions often violated by ANOVA and GLMs when handling non-negative data. Given the exploratory nature of the study and the wide range of variables considered here, we select the best-fitting model based on three well-estabilished criteria — AIC, BIC, and R-squared — while limiting the final model to a maximum of five predictors to ensure interpretability and reduce model complexity and overfitting. This approach combines the strengths of each metric, achieving a balance between model parsimony, fit quality, and predictive accuracy (all the code to reproduce the results are fully available in the OSF repository). The full model initially included all predictors, i.e., Demographic (Age, Education), Past infection (Covid-19), Attitudes toward physical touch (touching or being touched is intrusive [in general], pleasant [by professionals], unpleasant [by unknown people]), Resilience (Self-action Control, Positivity in tough situations), Reactance (Frustration and Anger due to freedom limitation), Trust in healthcare professions (Trust in Covid-19 Scientific Research and in Medical Guidelines), Negative emotional experience when using face masks (controlled, weak, scared, silly), Positive emotional experience when using face masks (brave, caring, strong, protected), Current use of face masks (open, closed environments), Covid-19-related fears (MAC-RF), Personality traits (negative affectivity, detachment, antagonism, disinhibition, psychoticism), Depression (PHQ-9), Generalised anxiety (GAD-7). Through iterative steps, predictors that did not improve the model, as assessed by the Akaike Information Criterion (AIC), Bayesian Information Criterion (BIC) and R-squared were removed. Via such a backward elimination process, we calculated the initial AIC and BIC of the full model and then fitting models by removing each predictor one at a time to calculate each relative AIC, BIC and R-squared [35,36]. The predictor whose removal reduced AIC and BIC, and increased R-squared the most was identified, and the final model was updated by removing it. This process continued until no further improvement was observed. The final model, obtained after backward elimination, included the selected predictors and was evaluated through various fit statistics: AIC, BIC, and R-squared, with 1044 observations. All models were fitted with the lme4 package [37] (S1 Table, S2 File).

## Results

### Descriptives

Sociodemographic data and descriptive analysis of the *Ad Hoc Socio-Demographic Interview* and psychological questionnaires (MAC-RF, PID-5-BF, GAD-7, PHQ-9) are summarized in Table 1 and Table 2.

**Table 1.** Demographic characteristics of participants.

Variable	M (sd)	n	%	Variable	n	%
Age				Educational level		
years	32.4 (12.1)			Lower Secondary	59	5.1
				Upper Secondary	468	40.7
Sex assigned at birth				Bachelor’s	307	26.7
Female		768	66.8	Master’s	250	21.7
Male		382	33.2	Postgraduate	12	1.0
Nationality				Covid-19 Vaccination		
Italian		1135	98.6	Yes	1121	97.4
Other		16	1.4	No	30	2.6
Relationship status				Past infection (Covid-19)		
Not in a relationship		815	70.8	Yes	697	60.7
In a relationship		336	29.2	No	451	39.3

**Table 2:** Descriptive analysis of the Ad Hoc Socio-Demographic Interview and psychological questionnaires (MAC-RF, PID-5-BF, GAD-7, PHQ-9).

Description		
	*Mean*	*SD*
*Attitudes toward physical touch*		
I often find touching or being touched an intrusive gesture	3.27	1.18
I find that physical contact with professionals is pleasant	3.77	1.02
I find it very unpleasant to have physical contact with unknown people	4	1.17
*Resilience*		
No matter what happens to me, I believe I can control my reactions	3.59	0.77
I believe I can grow well and in a positive way by facing difficult situations	3.61	0.81
*Reactance*		
I get frustrated when I can’t make free and independent decisions	4.04	0.94
I get angry when my freedom of choice is restricted	4.24	0.88
*Trust in Healthcare Professions*		
I trust scientific research and its findings	4.58	0.75
I trust the health professions and their indications	4.51	0.76
*Emotions experienced when wearing face masks*		
Controlled	1.96	1.32
Weak	1.43	0.91
Scared	1.44	0.90
Silly	1.51	1.03
Brave	2.15	1.24
Caring	4.11	1.14
Strong	2.61	1.29
Protected	4.07	1.10
*Covid-19-related fears*		
MAC-RF TOT	10.54	6.18
*Personality Traits*		
negative affectivity	5.71	2.85
detachment	3.82	2.70
antagonism	3.04	2.40
disinhibition	3.29	2.72
psychoticism	4.33	2.96
PID-5-BF TOT	20.19	10.45
*Anxiety Severity*		
GAD-7 TOT	7.22	4.82
*Depression Severity*		
PHQ-9 TOT	7.87	5.68

### Generalized mixed-effect modeling

**Fig 1:**
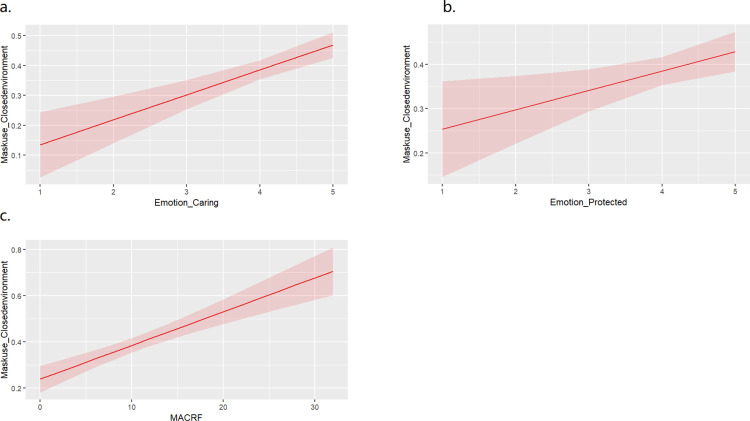
Effect plot with 95% Confidence Interval estimated by the logistic regression model predicting face mask-wearing in closed environments by a. emotion caring, b. emotion protected and c. Covid-19-related Fears.

The final logistic regression model predicting the use of the face mask in closed environments included the following five fixed factors: Feeling (Emotion) Caring, Feeling (Emotion) Protected, Feeling (Emotion) Strong, Feeling (Emotion) Brave and Covid-19-related Fears. Table 3 and Fig 1 display that the emotional experience related to face mask use shows an effect indicating that feeling caring, feeling protected, and the Covid-19-related fear levels increase the probability of wearing a face mask in closed environments.

**Table 3:** Estimated linear regression coefficients, 95% confidence intervals, and p-values of the selected model for each factor.

	**FACEMASK Closed Environment**		
*Predictors*	*Odds Ratios*	*CI*	*p*
(Intercept)	‒0.35	‒0.46– -0.23	**<0.001**
Emotion Caring	0.08	0.05– 0.12	**<0.001**
Emotion Protected	0.04	0.01–0.08	**0.011**
MACRF	0.01	0.01–0.02	**<0.001**
Emotion Strong	0.02	‒0.01–0.05	0.161
Emotion Brave	0.01	‒0.02–0.04	0.615
*Random Effects*			
σ^2^	3.29		
τ_00 sub_	136.64		
ICC	0.98		
N _sub_	1134		
Observations	4524		
Marginal R^2^/ Conditional R^2^	0.157/ 0.153		

## Discussion

On 5 May 2023, the WHO declared the global state of emergency for Covid-19 over. Three years after the start of the emergency, numerous studies have investigated individual adherence to protective equipment devices, enhancing our understanding of human compliance with healthy behaviours (e.g., [3,21,25,27]). The present study aimed to analyze how individual attitudes, namely demographic, attitudinal, psychological, and emotional factors, the latter linked to the experience of wearing a face mask, influenced the decision to still wear face masks when they were not formally required.

The regression model showed that fears related to Covid-19 and emotional experiences, linked to feeling caring and protected when wearing the face mask, play an important role in predicting face mask use.

Specifically, greater fears related to Covid-19 predicted higher face mask use, which is consistent with previous studies in the literature claiming that contamination fears predict greater safety behaviors [20,21,22]. Moreover, the high propensity to wear a face mask is associated with higher levels of anxiety and Covid-19-related fear. Previous research has shown that wearing a face mask is associated with risk perceptions during epidemics [38,39] and, specifically during the Covid-19 pandemic (e.g., [40]). In the present study the emotional experience of feeling caring and protected was found to be associated with higher mask use in line with the literature [12,13,14,15,16]. Other studies have investigated the specific emotional experience or physiological discomfort caused by wearing a face mask during the pandemic [6,8,10], but none when the face mask was no longer mandatory. In alignment with the above-mentioned studies, our findings indicated that feeling protected is associated with an enhanced willingness to wear a face mask.

The results of this study did not find any evidence that stable personality traits such as psychoticism or negative affectivity may be strong predictors of face mask use. As personality traits represent generalised behavioural models that actively respond to the context by implementing specific patterns, they have been studied in relation to the Covid-19 pandemic. Previous findings indicated an association between an increase in psychoticism traits and high levels of negative affectivity, as well as a greater likelihood of having Covid-19-related fears [41]. Instead, situational factors, such as the emotional experience associated with wearing face masks and fears of contagion appear to play a more critical role. Consistent with our findings from the regression analysis, a supplementary network analysis in the Supplementary Information (S3 Table, S4 File) replicated that increased use of face masks was correlated with an increase in positive emotional experiences. This highlights the need to promote health-protective behaviours by focusing on improving the situational emotional experience, rather than relying solely on personality traits. However, supplementary analysis also suggested that Covid-19-related fears were found to link personality traits, depression, and anxiety, with face mask-wearing. Increased use of face masks correlated with an increase in positive emotional experiences, which was subsequently associated with a decrease in negative emotions. Nevertheless, it remains possible that personality traits may play a more fundamental role in how individuals respond to enforced rules. That is, when face mask mandates are lifted, different factors may influence the decision-making process regarding face mask use.

The conclusions drawn from this study should be considered with a number of limitations. Firstly, it was not possible to assess all relevant individual attitudes within a single study. It would have been beneficial to include additional variables that have been shown to be associated with the choice to wear a face mask, such as endorsement of masculinity, political orientation, and beliefs about the effectiveness of face masks [13,42]. It should also be noted that the wearing of face masks was still mandatory in healthcare settings during the data collection period, which was not taken into account in the survey used in the study. However, the results of this research can be seen as an intriguing starting point for conducting the additional studies needed to further explore causality. Exploring a cross-validation strategy by training the model on a segment of the sample could have been a compelling option. This would have allowed an assessment of the robustness and transferability of the analysis results. Changing the set of variables could potentially lead to slightly different results. In order to tackle this problem, we have endeavored to include as comprehensive a set of variables as possible. However, we did not include many specific demographic variables, such as gender.

In terms of strengths, this study had a large sample size. In addition, to the best of our knowledge, this study is the first attempt to provide a framework for the many factors involved in the use of face masks in closed environments when they were no longer mandatory. Given the significant impact of the Covid-19 pandemic as a global health crisis, there is an urgent need to learn from this experience and improve our preparedness for future outbreaks. Investigating the use of face masks in closed and open environments, through research on emotional experiences linked to behavioural outcomes, can provide insights and data to inform public health strategies for effective management of emerging infectious diseases. Furthermore, investigating the reproducibility of these findings across different countries is an important avenue for future research, given the diverse impact of the Covid-19 pandemic on a global scale. Factors such as cultural nuances, healthcare systems, government responses, and socio-economic contexts can significantly shape individuals’ emotions and behaviour regarding the practice of face masks.

The clinical implications that our work may suggest are directed towards primary prevention of well-being. In particular, public health campaigns promoting the use of face masks should not only focus on the scientific evidence explaining how mask use prevents infection, but also place more emphasis on the direct relationship between the sense of caring and positive emotional responses associated with the use of face masks, such as feeling protected, strong and brave. Therefore, interventions that promote the positive emotional experience would weaken the association between face mask use and negative emotional responses such as feeling anxious and controlled.

## Supporting information

S1 Table Regression analysis.
The data source for the regression analysis https://doi.org/10.5061/dryad.fbg79cp3g.
(CSV)

S2 File Regression analysis script.
This is the.R script to run the regression analysis https://doi.org/10.5281/zenodo.14056914.
(R)

S3 Table Network analysis.
The data source for the additional network analysis https://doi.org/10.5281/zenodo.14056916.
(CSV)

S4 File Network analysis script.
The.R script to run the network analysis https://doi.org/10.5281/zenodo.14056916.
(R)

S1 Dtata (ZIP)
