# Supplementary material for: Wearing face masks when no longer mandatory: An exploratory study about attitudinal and psychological health factors in a large Italian sample
Source: PLoS One. 2025 Mar 4;20(3):e0314607. doi: 10.1371/journal.pone.0314607 (PMC11878940; doi:10.1371/journal.pone.0314607)
Supplement: S1 Dtata — (ZIP) [file pone.0314607.s005.zip › Supporting Information/robustness_check.pdf]

Robustness check via the network analysis

In order to jointly consider the weighted associations between variables and to understand the complex matrix of associations expected in our data collection, we chose network analysis as a tool to explore the weighted multivariate correlation between measures, by dealing with the complexity of distributed networks. Specifically, we used the 'bootnet' package (1) to estimate a model based on multiple subjects. It is noteworthy that network analysis is a set of techniques that are useful for the representation of multiple variables and the study of their relationships. The basic assumption is that studying the associations between a plausible number of variables offers better explanations of a complex phenomenon such as the link between attitude and behaviour. This is particularly true when comparing the network analysis approach to the traditional correlation studies that usually limit their investigation to two variables at a time. In fact, the network analysis organizes the entire data collection in a data matrix, thereby facilitating the representation of all the variables of interest as nodes and their relationships as lines (edges). In this way, the concept of a network becomes an analytical and operational tool that uses mathematical language and graph theory, to help us define plausible relationships between attitude and behavior. Of note, the initial fundamental step in interpreting the results of a network analysis is the centrality of the nodes. A node that has numerous and robust associations with other nodes is, therefore, more central within the network than other nodes. For each node, we estimated the Strength and the Expected Influence (EI) (2). We compared three different estimated networks (i.e., standard EBISglasso, a more conservative EBICglasso with a threshold, and unregularised estimation). We finally selected the estimated networks by conservative EBICglasso with a threshold. We did so given that the typical GLASSO algorithm sometimes fails to retrieve the correct model and gives rise to small false-positive edges, which in turn affects the replicability of small edges over different empirical samples (3).

1. Epskamp S, Fried EI. Package "bootnet". R package version, 1. 2020.

2. Robinaugh DJ, Millner AJ, McNally RJ. Identifying highly influential nodes in the complicated grief network. J Abnorm Psychol. 2016;125(6):747–57.

3. Borsboom D. Possible Futures for Network Psychometrics. Psychometrika. 2022 Mar 1;87(1):253–65.

Network Analysis

Figure 1 shows the estimated network of weighted relationships among the variable Socio-demographic (Age, Education), Past infection (Covid-19), Attitudes toward physical touch (1 and 2), Resilience (2,3), Reactance (1,2), Trust in healthcare professions (1,2), Negative emotional experience when using face masks (controlled_mask, weak_mask, scared_mask, silly_mask), Positive emotional experience when using masks (brave_mask, caring_mask, strong_mask, protected_mask), Current use of face masks (open, closed environments), Covid-19-related fears (MAC-RF), Personality traits (neg affectivity, detachment, antagonism, disinhibition, psychoticism), Generalized anxiety (GAD-7), Depression (PHQ-9). First, it emerges that within our sample, Socio-demographic variables (yellow nodes in the network) as well as a past Covid-19 infection do not show any association with any other nodes in the network. Similarly, differences in Resilience, Reactance, Trust in healthcare professions, and attitudes toward physical touch do not show any statistically significant associations within our network analysis. Secondly, strong and positive associations emerge between personality traits (blue nodes) and indicators of generalized anxiety

(GAD-7) and Depression (PHQ-9). In particular, Negative Affectivity shows a statistically positive association with Covid-19-related fears (MAC-RF). In this sense, Covid-19-related fears (MAC-RF) connect Personality traits and Depression (PHQ-9), including anxiety levels (GAD-7), with the use of face masks in closed environments. It is important to note in the present network analysis that an increase in face mask use in closed environments corresponds to an increased probability of wearing face masks in open environments and the emotional experience and motivations associated with this use seem to be positive (brave, caring, strong and protected), which in turn are negatively correlated with negative emotional experience associated with face mask-wearing (controlled, weak, scared, silly).

Figure 1. The estimated network represents the partial correlation matrix between Demographic (A1-2: age, Education), Past infection (B1: Covid-19), Attitudes toward physical touch (C1-3: 1,2 and 3), Resilience (D1-2: Self-action Control, Positivity in tough situations), Reactance (E1-2: Frustration and Anger due to freedom limitation), Trust in healthcare professions (F1-2: Trust in Covid-19 Scientific Research and in Medical Guidelines), Negative emotional experience when using face masks (G1-4: controlled, weak, scared, silly), Positive emotional experience when using face masks (G5-8: brave, caring, strong, protected), Current use of face masks (H1-2: open, closed environments), Covid-19-related fears (I1: MAC-RF), Personality traits (J1-5: negative affectivity, detachment, antagonism, disinhibition, psychoticism), Depression (L1: PHQ-9), G eneralised anxiety (K1: GAD-7). Positive associations are depicted with green lines, whereas negative associations are depicted with red lines. The size and color density of the lines (edges) vary to reflect the varying strength of the relationship between the variables; the edges are non-directional as the data is represented as bivariate partial correlations between the variables. Please note that darker and thicker lines indicate stronger correlations.

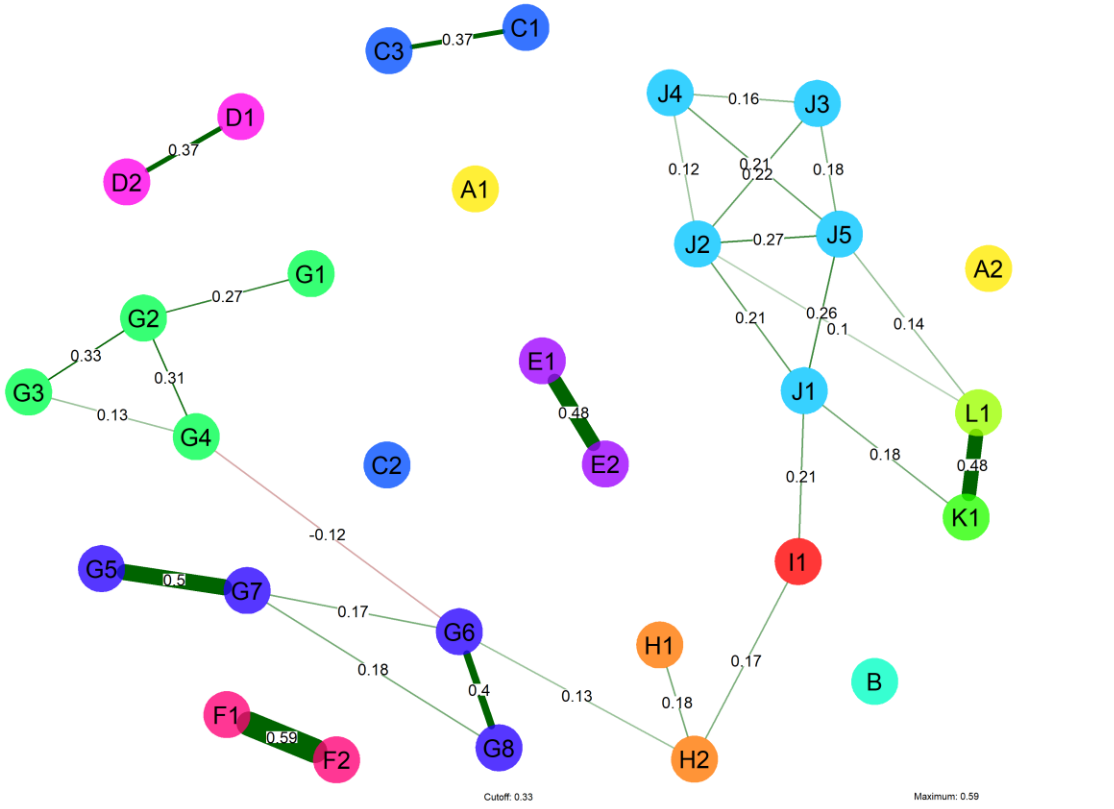

Demographics
A1: Education
A2: Age

Past Covid Infection
B

Attitude Towards Physical Touch
C1: Touching
C2: Physical contact with Professionals
C3: Physical contact with uknowns

Resilience
D1: Self-action Control
D2: Positivity in Tough Situations

Reactance
E1: Frustration due to Freedom Limitation
E2: Anger due to Freedom Limitation

Trust in Healthcare Professions
F1: Covid-19 Scientific Research
F2: Covid-19 Medical Guidelines

Emotion wearing face masks
G1: Controlled
G2: Weak
G3: Scared
G4: Silly

Emotion wearing face masks
G5: Brave
G6: Caring
G7: Strong
G8: Protected

Current use of face masks
H1: Open Environment
H2: Closed Environment

Covid Related Fears (MAC-RF)
I1

Personality Traits
J1: Negative Affectivity
J2: Detachment
J3: Antagonism
J4: Disinhibition
J5: Psychoticism

Depression
L1

General Anxiety
K1

Figure 2 below shows that the conservative GLASSO indicates the centrality of the node Psychoticism when compared to all other nodes within the network. In particular, in our network analysis, Psychoticism showed a distinguishable Strength that refers to the intensity or importance of connections between nodes, and higher Expected influence that estimates the potential impact of such a node in spreading information or influencing other nodes within our network. In addition, Closeness, which measures how easily information flows through the network by calculating the distance between nodes, indicates that the current use of the face mask in a closed environment, feeling weak while using it, and the presence of Covid-19-related fears are the nodes that facilitate the connection between other nodes of the network. In addition, Betweenness, which quantifies the extent to which a node acts as a bridge connecting other nodes, suggests that feeling caring while using the face mask, the current use of the face mask in a closed environment, the Covid-19-related fears and Negative affectivity are candidates nodes for explaining the connection among the whole network.

Lastly, to better interpret the network analysis results, we estimated the stability of the edge-weight parameters and the edge-weight accuracy. As shown by the bottom of Figure 2, the small gray area indicates that the network analysis offered a stable estimation.

Figure 2. Strength, Closeness, Betweenness and Expected influence. The red docs give the edge weights of the network, and the gray area is the 95% CI around the weights. In both graphs, the x-axis indicates the estimated strength for each node.

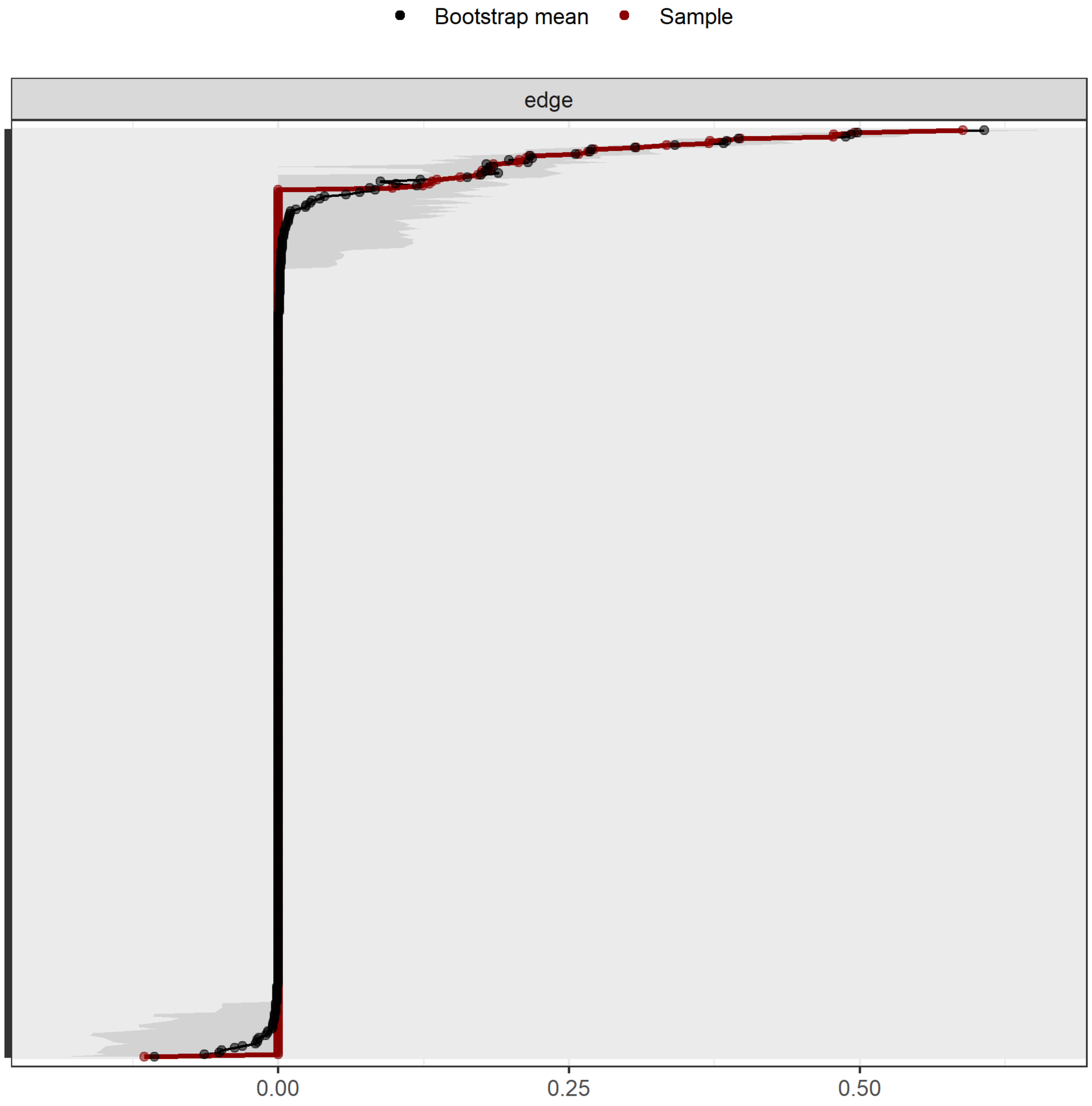
Bootstrap mean
Sample
edge
0.00
0.25
0.50

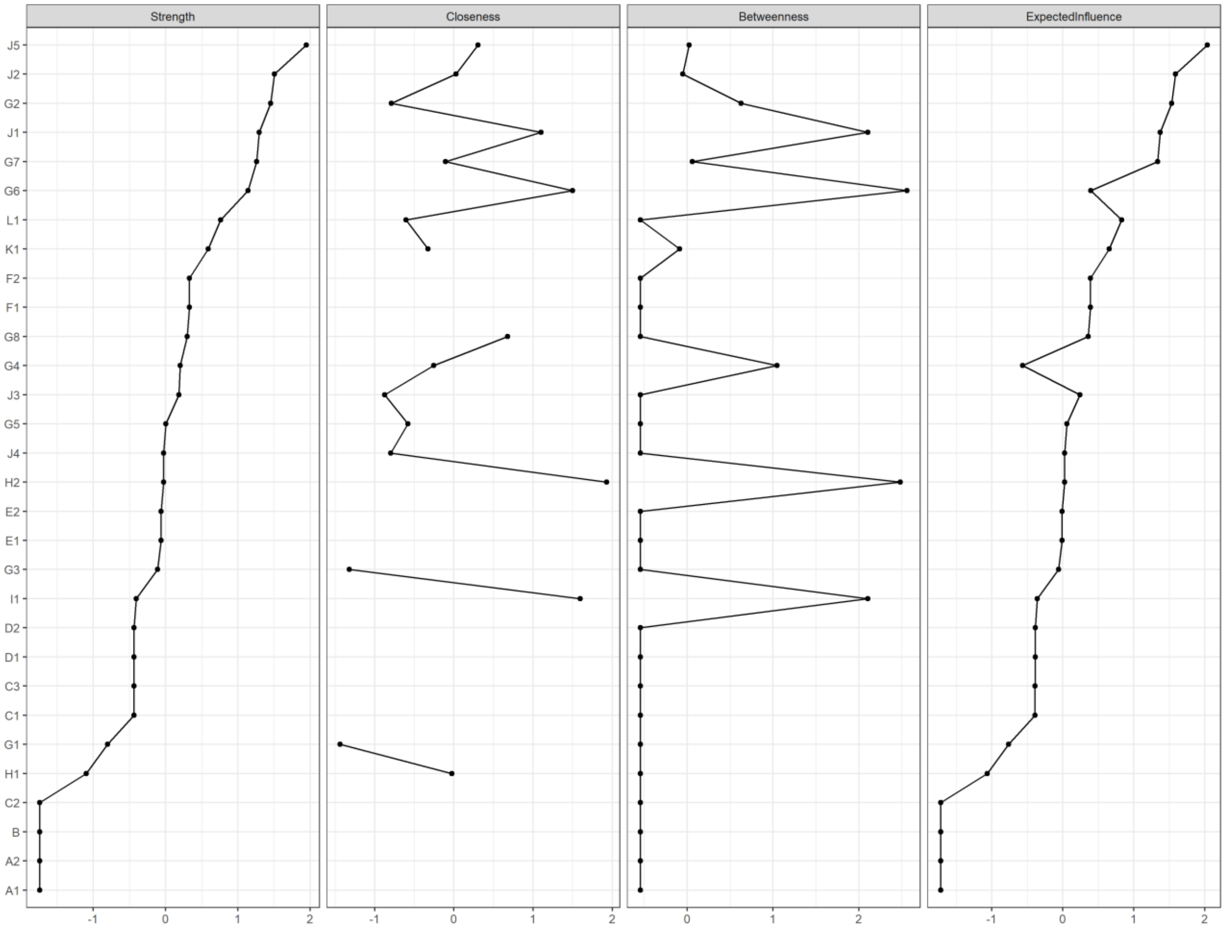

Strength
Closeness
Betweenness
ExpectedInfluence
J5
J2
G2
J1
G7
G6
L1
K1
F2
F1
G8
G4
J3
G5
J4
H2
E2
E1
G3
I1
D2
D1
C3
C1
G1
H1
C2
B
A2
A1
-1
0
1
2

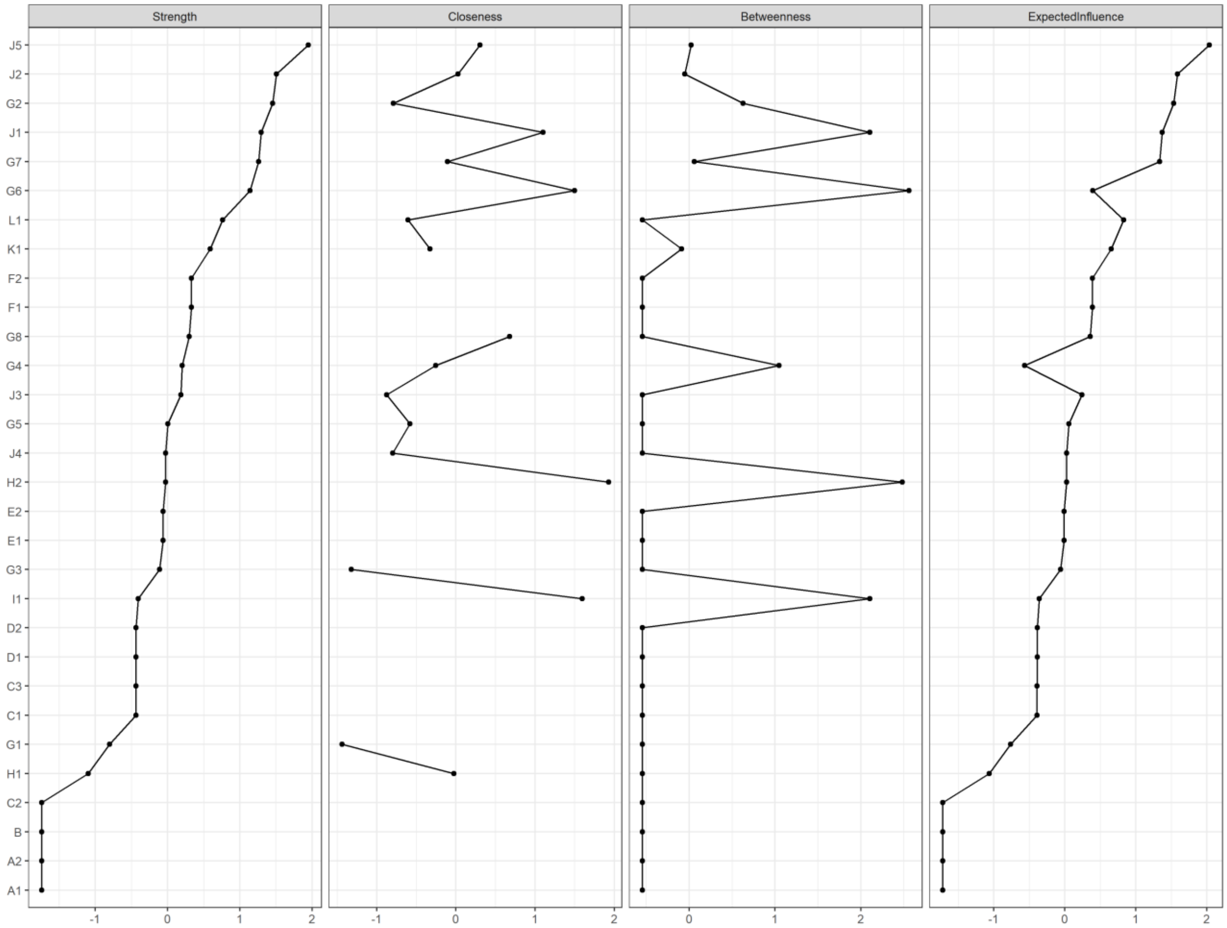

Strength
Closeness
Betweenness
ExpectedInfluence
J5
J2
G2
J1
G7
G6
L1
K1
F2
F1
G8
G4
J3
G5
J4
H2
E2
E1
G3
I1
D2
D1
C3
C1
G1
H1
C2
B
A2
A1
-1
0
1
2
